# Addressing complexities in β-thalassemia care: a case series from a resource-limited setting

**DOI:** 10.1097/MS9.0000000000002471

**Published:** 2024-08-14

**Authors:** Pratik Adhikari

**Affiliations:** B.P. Koirala Institute of Health Sciences, Dharan, Nepal

**Keywords:** beta-thalassemia, case series, community engagement, management challenges, resource-limited settings

## Abstract

**Introduction and importance:**

β-thalassemia is a hereditary blood disorder with a global prevalence, presenting diagnostic and management challenges, particularly in regions with high consanguinity rates. Diagnostic methods include clinical assessments, genetic testing, and hemoglobin electrophoresis. Treatment typically involves transfusions and chelation therapy, with gene therapy showing promise. This case series emphasizes the need for tailored care strategies and global health initiatives to improve outcomes for β-thalassemia patients worldwide.

**Methods:**

This case series involves five patients from rural Nepal presenting various β-thalassemia manifestations. The cases highlight the challenges in diagnosis and management in resource-limited settings. Data were collected through clinical assessments, laboratory investigations, and follow-ups. Each patient’s medical history, presentation, and treatment regimen were documented.

**Outcomes:**

The cases underscore the importance of regular follow-ups, community engagement, and personalized treatment strategies tailored to genetic profiles. Key findings include the necessity for consistent transfusion schedules, iron overload monitoring, and managing complications associated with β-thalassemia. Enhanced education and healthcare collaboration were noted as critical for optimizing care and outcomes in resource-limited settings.

**Conclusions:**

Managing β-thalassemia in resource-limited settings demands timely intervention, regular monitoring, and community involvement. Enhanced healthcare collaboration, access to advanced diagnostic tools, and tailored treatment strategies are paramount in addressing the unique challenges of β-thalassemia. These measures are essential for ensuring an improved quality of life for affected individuals in such regions.

## Introduction

Highlights

- This case series presents five patients with β-thalassemia from rural Nepal, highlighting the diagnostic and management challenges in resource-limited settings.
- Regular blood transfusions and chelation therapy were crucial in managing anemia and preventing complications, with consistent follow-ups leading to improved outcomes.
- The importance of community engagement, patient education, and personalized treatment strategies tailored to genetic profiles was emphasized to optimize care in resource-limited areas.
- The study underscores the need for enhanced healthcare collaboration and access to advanced diagnostic tools to improve the quality of life for β-thalassemia patients globally.

β-thalassemia is a hereditary blood disorder that presents significant challenges in hematology, affecting around 1 in 100 000 births worldwide^1^. However, its prevalence is notably higher in regions with increased rates of consanguinity, such as Mediterranean countries, parts of South Asia, and the Middle East. The concentration of β-thalassemia cases in these areas creates complexities in diagnosis and management due to varying healthcare infrastructure and resource availability. Socio-economic factors also play a crucial role, impacting access to advanced diagnostic tools, genetic counseling, and specialized treatments. Disparities in healthcare resources between developed and developing regions further compound the challenges associated with β-thalassemia diagnosis and care, necessitating global health initiatives to address the unequal burden of this disorder.

Diagnosing β-thalassemia involves clinical assessments, complete blood count (CBC) analysis, hemoglobin (Hb) electrophoresis, and genetic testing to identify specific mutations. The severity of the condition ranges widely, from mild carriers to individuals requiring frequent blood transfusions. Management strategies encompass a multidisciplinary approach, including transfusions to manage anemia and chelation therapy to mitigate iron overload from transfusions. β-thalassemia disrupts hemoglobin production, resulting in anemia and a range of clinical symptoms. Mutations in the β-globin chains of hemoglobin cause an imbalance between alpha and beta chains, leading to the premature breakdown of red blood cells (RBCs)^2^. The genetic diversity of β-thalassemia mutations influences disease severity, treatment response, and clinical management complexity, emphasizing the need for personalized care strategies tailored to each patient’s genetic profile. While gene therapy and stem cell transplantation hold promise for a permanent cure, challenges remain due to the disorder’s genetic diversity and global distribution^3^. Here, we describe a case series of five patients who presented to a tertiary healthcare center with features suggestive of β-thalassemia, admitted, and managed in the ward. This report is aligned with the preferred reporting of case series in surgery (PROCESS) 2020 guidelines^4^.

## Case series

### Case 1

A 6-year-old boy from a rural village in rural Nepal presented with symptoms of fatigue, pallor, and shortness of breath, which had been ongoing for several months. He was diagnosed with severe microcytic hypochromic anemia, indicative of beta-thalassemia major. Physical examination revealed signs of growth retardation, pallor of conjunctiva and nail beds, and early dental abnormalities. Hemoglobin electrophoresis confirmed the diagnosis, leading to the initiation of regular blood transfusions every 3 weeks. Chelation therapy with deferasirox was started to manage iron overload due to repeated transfusions. However, the patient failed to follow up regularly despite initial treatment and diagnosis. This case highlights the challenges in managing chronic conditions like beta-thalassemia in remote areas, where healthcare access and awareness are limited. Regular follow-up visits are crucial for monitoring hemoglobin levels, managing iron overload, and assessing growth and development milestones. Proper counseling and community engagement play a vital role in ensuring patients adhere to treatment plans and receive necessary support, including access to blood supplies. Timely intervention, regular monitoring, and community involvement are essential for optimizing outcomes and preventing complications associated with beta-thalassemia. The scarcity of reported cases from such regions underscores the need for increased awareness, early diagnosis, and comprehensive management strategies for genetic blood disorders in resource-limited settings like rural Nepal.

### Case 2

A 9-year-old boy from rural Nepal with a history of recurrent febrile episodes was diagnosed with beta-thalassemia at age 5. He presented with symptoms of fatigue, delayed growth, and jaundice, necessitating three blood transfusions in the last 6 months due to worsening anemia. Physical examination findings and laboratory tests confirmed beta-thalassemia major with characteristic features such as pallor, icterus, microcytic anemia, and elevated ferritin levels. Genetic testing revealed homozygosity for a rare beta-thalassemia mutation. Management involved regular blood transfusions every 3 weeks and chelation therapy with deferasirox to manage iron overload. Despite challenges in access to specialized care and blood products in rural areas of Nepal, consistent treatment resulted in improved symptoms, stable hemoglobin levels, and appropriate growth. Enhanced community-based education and healthcare collaboration are crucial for optimizing care and outcomes for beta-thalassemia patients in such settings.

### Case 3

A 22-year-old female from rural Nepal, diagnosed with beta-thalassemia since childhood and undergoing regular blood transfusions, presented with acute left hip pain of two weeks’ duration. There was no history of trauma reported. On examination, localized tenderness over the left hip joint and a restricted range of motion were noted. No signs of neurological deficits were found, and peripheral pulses were intact. Laboratory investigations revealed low levels of factor VII and factor XIII. Imaging studies, including X-ray and ultrasound Doppler of the left lower limb, ruled out bony abnormalities and deep vein thrombosis, respectively. However, an MRI of the left hip showed heterogeneous marrow signal intensity with ill-defined cystic changes in the proximal femur metaphysis, suggestive of iron deposition and red marrow conversion seen in beta-thalassemia patients. Localized complex subperiosteal fluid collections were observed along the left femur, indicative of hematomas. Ultrasound-guided aspiration confirmed the presence of subperiosteal hematomas. The patient was managed conservatively with pain management and regular follow-ups with the hematology and orthopedics teams. The case underscores the importance of vigilant monitoring for musculoskeletal complications in beta-thalassemia patients, especially those with iron overload, emphasizing the need for multidisciplinary care in resource-limited settings.

### Case 4

A 6-year-old boy from rural Nepal, born to nonconsanguineous parents, presented with a history of recurrent blood transfusions (total of four) and occasional episodes of jaundice since infancy. On examination, the child exhibited growth failure, thalassemic facies, splenomegaly, hepatomegaly, and marked pallor. His first transfusion was administered at the age of 2 years. Laboratory investigations revealed a hemoglobin level of 6.2 g/dl, MCH of 20.8 pg, MCV of 52.1 fL, MCHC of 40.0 g/dl, and RDW of 27.5%. Peripheral smear analysis displayed anisopoikilocytosis, target cells, occasional nucleated RBCs, and fragmented cells. Hemoglobin electrophoresis demonstrated HbA at 30%, HbF at 40.2%, HbA2 at 3.8%, and HbE at 25%. Based on clinical findings and test results, a diagnosis of HbE β+-thalassemia was established. The parents were counseled regarding the diagnosis and advised to undergo electrophoresis for carrier status evaluation and genetic counseling for future pregnancies. A trial of hydroxyurea was recommended for the patient to assess its therapeutic benefits. The case highlights the challenges of managing beta-thalassemia in resource-limited settings like rural Nepal, emphasizing the importance of early diagnosis, genetic counseling, and exploring treatment options like hydroxyurea to improve clinical outcomes.

### Case 5

**Figure 1:**
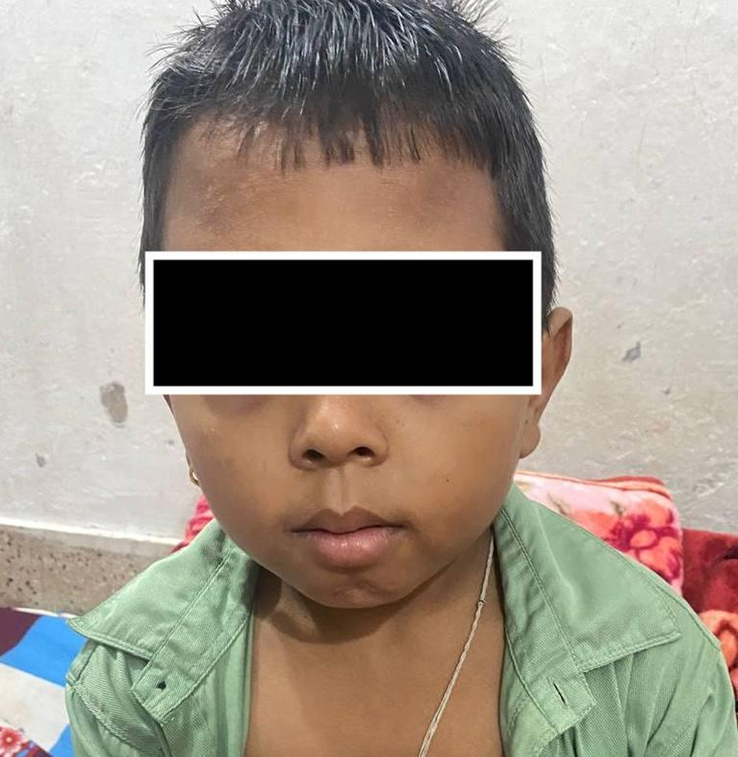
A young child with beta-thalassemia exhibiting common facial changes such as a prominent forehead, flattened nasal bridge, puffy cheeks, and a slightly protruded upper jaw.

A 10-year-old boy from rural Nepal presented with chronic fatigue, shortness of breath on exertion, and facial abnormalities since adolescence. As shown in Figure 1, the boy exhibits common facial changes associated with beta-thalassemia, including a prominent forehead, flattened nasal bridge, puffy cheeks, and a slightly protruded upper jaw, which are typical due to bone marrow expansion. Upon examination, he displayed characteristic ‘Chipmunk facies’, delayed puberty, short stature, and mild splenomegaly. Skull X-ray revealed the classic ‘crew-cut’ appearance indicative of extramedullary hematopoiesis. Hemoglobin electrophoresis showed elevated fetal hemoglobin (HbF) levels at 95% and alpha2 hemoglobin at 2.3%. Despite no history of transfusions since childhood, the diagnosis of β-thalassemia intermedia was confirmed based on clinical presentation and genetic mutation analysis, which revealed the IVSI 5 (G→C) mutation. Additionally, a positive direct Coombs test suggested autoimmune hemolytic anemia coexistence. Treatment involved hypertransfusion and prednisolone therapy, leading to stabilization of hemoglobin levels around 9.5 g/dl. The patient is awaiting a human leukocyte antigen-matched stem cell transplant from his unaffected younger sibling. This case highlights a rare occurrence of β-thalassemia intermedia with advanced extramedullary hematopoiesis in rural Nepal, underscoring the importance of genetic testing and tailored treatment approaches for optimal patient management.

All the cases are summarized in Table 1.

**Table 1:** Summary of all cases.

Case	Age (years)	Presentation	Physical findings	Lab investigations and findings	Treatment	Conclusion
1	6	Fatigue, pallor, and shortness of breath	Growth retardation, pallor, and early dental abnormalities	Severe microcytic hypochromic anemia, Hb electrophoresis confirmed beta-thalassemia major	Regular blood transfusions, chelation therapy with deferasirox	Challenges in managing chronic conditions in remote areas, emphasizing regular follow-ups and community support
2	9	Fatigue, delayed growth, jaundice	Pallor, icterus, microcytic anemia, elevated ferritin levels	Beta-thalassemia major confirmed, homozygosity for rare mutation	Regular blood transfusions, chelation therapy with deferasirox	Consistent treatment resulted in improved symptoms and stable hemoglobin levels
3	22	Acute left hip pain	Localized tenderness over left hip, restricted range of motion	Low levels of factor VII and factor XIII, MRI showed iron deposition	Pain management, regular follow-ups with hematology and orthopedics	Importance of monitoring musculoskeletal complications in thalassemia, need for multidisciplinary care
4	6	Recurrent blood transfusions, jaundice	Growth failure, thalassemic facies, splenomegaly, hepatomegaly, marked pallor	Severe microcytic hypochromic anemia, hemoglobin electrophoresis confirmed HbE β+-thalassemia	Hydroxyurea trial recommended, genetic counseling	Challenges of managing thalassemia in resource-limited areas, early diagnosis is crucial
5	10	Chronic fatigue, shortness of breath, facial abnormalities	“Chipmunk facies,” delayed puberty, short stature, mild splenomegaly	Elevated HbF levels, IVSI 5 (G→C) mutation, positive Coombs test	Hypertransfusion, prednisolone therapy, awaiting stem cell transplant	Rare occurrence of β-thalassemia intermedia with advanced extramedullary hematopoiesis, need for tailored treatment

## Follow-up and outcomes

All patients were followed up regularly to monitor hemoglobin levels, manage iron overload, and assess growth and development. Complications such as iron overload, musculoskeletal issues, and the need for genetic counseling were addressed through a multidisciplinary approach. Regular blood transfusions and chelation therapy were crucial in managing anemia and preventing complications. Enhanced community engagement and education were essential in ensuring patient adherence to treatment plans. Despite the challenges, consistent follow-up and personalized treatment strategies led to improved outcomes and better quality of life for the patients. No major adverse events were reported during the follow-up period, highlighting the effectiveness of the management strategies employed.

## Discussion

The prevalence of β-thalassemia is notably high in regions such as the Middle East, Central Asia, the Indian Subcontinent, and the Far East, with Plasmodium falciparum malaria contributing to elevated case numbers in these areas^5^. The disorder often manifests as severe anemia and skeletal abnormalities, alongside specific clinical and radiological symptoms^6^. Persistent anemia can lead to cerebral hypoxia, increasing the risk of ischemic events and strokes. Patients not receiving adequate treatment often exhibit more traditional symptoms^7,8^. Acute neurological complications in β-thalassemia patients include cerebral ischemia, spinal cord fractures, and compression from extramedullary hematopoietic malignancies^9,10^.

Our case series shares similarities with other studies conducted in rural settings. For instance, Ansari *et al*.^11^ documented a case of right hemiparesis in a 25-year-old female with β-thalassemia major who had undergone frequent blood transfusions until the age of nine. Comparatively, our case series includes a 12-year-old female with β-thalassemia major who experienced left hemiparesis at the age of five, illustrating the variability in neurological complications and the critical role of regular transfusions in managing such conditions. However, stroke in thalassemia is attributed not only to a hypercoagulable state but also to cardioembolic events and large vessel thrombosis^12^.

Regular transfusions are essential in mitigating the abnormal aggregation of thalassemic red blood cells (RBCs), reducing the risk of thromboembolic events, which are prevalent in patients with limited transfusion history^13^. However, transfusion therapy poses the risk of iron overload, which can deposit in the brain, exacerbating oxidative stress and neuronal damage. Chelating agents like deferoxamine and deferasirox are crucial in managing these complications^14^. Despite chelation therapy, growth retardation, and delayed sexual maturation are persistent issues, highlighting the need for comprehensive care strategies. Additionally, adults with human homeostatic iron regulator protein (HFE)-associated hereditary hemochromatosis often experience cardiac (dilated cardiomyopathy, pericarditis), hepatic (chronic hepatitis, fibrosis, cirrhosis), and endocrine gland (diabetes mellitus, hypoparathyroidism, hypothyroidism, hypopituitarism, adrenal insufficiency) complications^14^.

Comparing our findings with similar studies in rural settings, such as those conducted in India and Bangladesh, underscores the need for enhanced community-based education, healthcare collaboration, and improved access to specialized care and blood products^15^. These interventions are vital for optimizing care and outcomes for β-thalassemia patients in resource-limited settings.

## Limitations and challenges

The study faced several limitations inherent to resource-limited settings. These include inconsistent follow-ups due to limited healthcare access, inadequate infrastructure for advanced diagnostic tools, and challenges in ensuring regular transfusions and chelation therapy. Additionally, socio-economic barriers and lack of awareness among patients and their families further compounded these challenges.

## Future directions

Future work should focus on developing robust healthcare networks and community engagement programs to enhance patient adherence to treatment regimens. Implementing telemedicine for regular follow-ups and genetic counseling could also mitigate the challenges posed by geographic and resource constraints. Furthermore, exploring affordable and accessible treatment alternatives, such as hydroxyurea therapy, could improve clinical outcomes in such settings.

By addressing these limitations and focusing on targeted interventions, we can enhance the quality of care for β-thalassemia patients in resource-limited settings, ultimately improving their quality of life and long-term outcomes.

## Conclusion

Our case series demonstrates the diverse clinical presentations and management challenges of beta-thalassemia in Nepal. We encountered cases such as β-thalassemia intermedia with advanced extramedullary hematopoiesis and unique genetic mutations. Despite resource limitations, we achieved promising outcomes through timely diagnosis, multidisciplinary care, and tailored treatment approaches. Our experiences emphasize the need for increased awareness and early genetic testing. We also found that collaborative healthcare strategies are essential for optimizing outcomes in patients with beta-thalassemia in resource-limited settings. Enhanced community-based education and healthcare collaboration are crucial in managing these patients effectively. Our findings highlight the importance of continuous efforts to improve healthcare infrastructure and access to specialized care in such settings.

## Ethical approval

There is no need for ethical approval for case series according to the local ethical guidelines.

## Consent

Written informed consent was obtained from the patients for publication and any accompanying images. A copy of the written consent is available for review by the Editor-in-Chief of this journal on request.

## Source of funding

Not applicable.

## Author contribution

PA led the drafting of the original manuscript, provided resources, and conducted review and editing.

## Conflicts of interest disclosure

The authors declare that they have no conflicts of interest.

## Research registration unique identifying number (UIN)

This is a case report involving a human subject, so registration of research study was done.

1. Name of the registry: Researchregistry.com.
2. Unique identifying number or registration ID: researchregistry10245.
3. Hyperlink to your specific registration (must be publicly accessible and will be checked): https://www.researchregistry.com/browse-the-registry#home/

## Guarantor

Pratik Adhikari is the guarantor of the study.

## Data availability statement

The datasets supporting the conclusions of this article are included within the article.

## Provenance and peer review

Not commissioned or externally peer-reviewed.
